# Mumps Encephalitis in a Measles Rubella (MR)-Vaccinated Child: A Rare but Preventable Complication

**DOI:** 10.7759/cureus.85318

**Published:** 2025-06-04

**Authors:** Sania Shahid, Fathimathul Henna, Jarisha Ali, Imran Asad, Sumbal Riaz, Mitra A Ghafoor zeyaei

**Affiliations:** 1 Pediatric Emergency Medicine, Al Jalila Children's Speciality Hospital, Dubai, ARE; 2 Medicine, Dubai Medical College for Girls, Dubai, ARE

**Keywords:** mmr vaccine, mr vaccine, mumps encephalitis, mumps epidemiology, mumps vaccine, pediatric neurology

## Abstract

Mumps is a vaccine-preventable disease caused by the mumps virus, mainly affecting the salivary glands. It can cause serious complications, including mumps encephalitis. Despite widespread immunization practices, mumps outbreaks persist among both vaccinated and unvaccinated populations, raising issues of concern regarding herd immunity. Mumps encephalitis, although rare, can produce serious complications warranting prompt diagnosis and management.

Here, in this case report, we present a five-year-old girl who presented with fever, bilateral parotid swelling, and a single episode of abnormal movement in the form of dystonia associated with fixed gaze and disorientation. The patient had received two doses of the measles rubella (MR) vaccine as per India’s universal immunization program (UIP). Laboratory investigations showed high amylase levels, positive serum mumps IgM, and cerebrospinal fluid pleocytosis; all indicative of mumps encephalitis. Brain imaging was normal, excluding other neurological causes. Initial empirical antibiotics and acyclovir were discontinued following the diagnosis of mumps encephalitis. The patient showed improvement within 48 hours of supportive treatment.

This case highlights the importance of considering mumps encephalitis in pediatric patients with mumps showing neurological symptoms. It also demonstrates the public health impact of omitting the mumps vaccine from India’s UIP and the major role of the mumps, measles, and rubella (MMR) vaccination in preventing such complications.

## Introduction

Mumps is a vaccine-preventable viral disease caused by the mumps virus, characterized by parotitis, fever, and malaise [1]. Widespread complications like orchitis, pancreatitis, and neurological complications like meningitis and encephalitis may occur [2]. Despite extensive vaccination programs, mumps outbreaks persist in vaccinated and undervaccinated populations, emphasizing the difficulties in achieving herd immunity [3,4].

Neurological complications, like mumps encephalitis, arise in roughly 1% to 10% of cases, indicating a rare yet severe consequence of the disease [5]. Mumps-associated encephalitis is believed to develop from primary viral attack of the central nervous system (CNS) or immune-mediated post-infectious mechanisms, frequently resulting in varied clinical outcomes ranging from mild confusion to coma [6]. Although mortality rates have reduced since the post-vaccination era, morbidity, including seizures, cognitive impairment, and prolonged neurological sequelae, remains a concern [7].

This report presents the case of mumps encephalitis diagnosed in a previously healthy five-year-old girl who primarily showed symptoms of fever, parotid swelling, and dystonic movements associated with disorientation. The diagnosis is supported by increased amylase levels, positive mumps serology, and analysis of cerebrospinal fluid, witha negative bacterial and viral encephalitic panel ruling out other causes. This case highlights the need to consider viral encephalitis in pediatric patients showing neurological symptoms and parotitis, regardless of the absence of confirmatory imaging findings.

## Case presentation

A five-year-old previously healthy girl presented to the emergency department with complaint of two-day history of fever, flu-like symptoms, and one episode of abnormal movement in the form of dystonia associated with fixed gaze and disorientation. The illness started with fever, cough, and overall discomfort, then progressed to painful bilateral parotid swelling. She was evaluated at an outpatient clinic where she was found to be febrile with a temperature of 38.5°C, normal C-reactive protein (CRP) 2.3 mg/L (reference :0-5 mg/L), leukopenia WBC 1.28 × 10³/uL initially which changed to 5.4 × 10³/uL(reference range: 5.0-15.0 10^3/uL), and normal hemoglobin and platelets. She received two doses of oral amoxicillin before presenting to the emergency department with acute neurological symptoms.

On the day of admission, the patient had a three-minute episode of unusual movement manifested by sudden fearfulness, screaming, dystonia, limb stiffening, and fixed gaze without post-ictal drowsiness, cyanosis, or incontinence. She continued to have fever and intermittent confusion, disorientation, and restlessness in the emergency department. The patient’s immunization card confirms that the patient received two doses of measles rubella (MR) vaccine as part of India’s immunization program. A history of sick contact was reported, as her younger sibling had recent upper respiratory symptoms.

**Figure 1:**
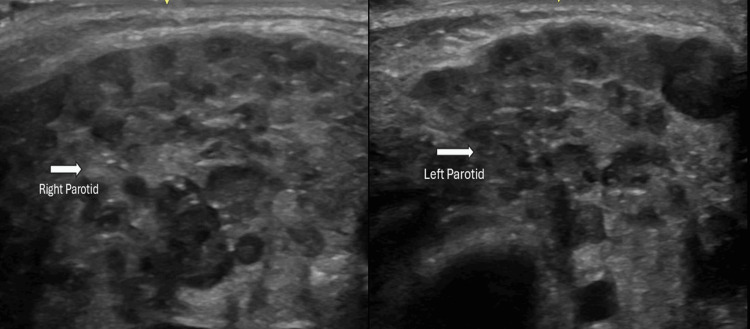
Bedside point of care ultrasound showing right and left parotid swelling

**Figure 2:**
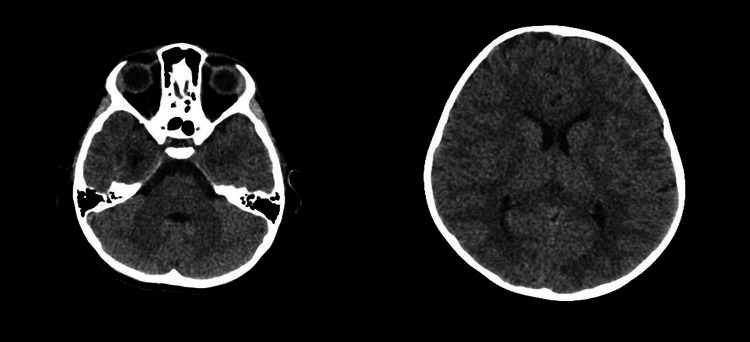
The CT of the brain is revealed to be normal (no evidence of intracranial bleeding or intracranial hypertension)

Initial examination revealed fever (temperature 38.1°C maximum reaching to 38.2°C) as shown in Table 1 and bilateral parotid swelling that was confirmed on bedside ultrasound (Figure 1). Neurological examination during the episodes revealed no focal deficits, normal gait, negative meningeal signs, normal power, tone, and reflexes. Computed Tomography (CT) of the brain was normal, ruling out hemorrhage or intracranial hypertension as shown in Figure 2.

**Table 1:** Vitals of the patient on presenting to the emergency department

Vitals	At 20:00	At 20:30	At 20:45	At 21:05	At 22:00
Temperature (tympanic)	37.7(99.9)	38.1 (100.6)	38 (100.4)	38.2 (100.8)	37.6 (99.7)
Pulse rate (beats/min)	100	129	113	114	122
Saturation (in room air)	98%	100%	98%	99%	99%
Blood pressure (mmHg)	93/67	98/66	101/69	94/68	99/67
Respiratory rate (breaths/min)	20	20	20	25	22

Laboratory investigations showed mildly elevated CRP 16.3 (reference range: 0-5 mg/L); normal procalcitonin 0.30 ng/mL (reference:<0.5 ng/mL), suggesting a nonbacterial cause. The serum amylase was markedly elevated (849 U/L; reference range: 8-96 U/L), consistent with parotitis. The serum mumps IgM was reported to be positive, thereby confirming acute mumps infection (Table 2).

**Table 2:** Laboratory Investigations on admission CRP: C-reactive protein,

Laboratory investigations	Result	Reference range
CRP	16.3 mg/L	0-5 mg/L
Procalcitonin	0.30 ng/mL	< 0.5 ng/mL
WBC count	5.4 × 10³/uL	5.0 - 15.0 × 10³/uL
Neutrophil absolute	4 × 10³/uL	1.5- 8.0 × 10³/uL
Lymphocyte absolute	1.2 × 10³/uL	6.0-9.0 × 10³/uL
Monocyte absolute	0.2 × 10³/uL	0.2-1.0 × 10³/uL
Eosinophils absolute	0	0.1-1.0 × 10³/uL
Basophils absolute	0.01 × 10³/uL	0.0-0.10 × 10³/uL
Hemoglobin	12.7 g/dL	11.1-14.1 g/dL
Platelets	228 × 10³/uL	200-490 × 10³/uL
Serum amylase	849 U/L (High)	8-96 U/L
Sodium (Na)	133 mmol/L (Low)	136-145 mmol/L
Bicarbonate (HCO3)	15.0 mmol/L (Low)	17-27 mmol/L
Glucose (random)	85 mg/dL	60-100 mg/dL
Mumps virus IgM	Positive	Negative
Mumps virus IgG	Immune	Immune index

Lumbar puncture revealed mild cerebrospinal fluid (CSF) pleocytosis (WBC 11 x 106/L; reference range: 0-7 10^6/L) with normal protein (11mg/L; reference range: 15-40 mg/dL). Multiplex CSF polymerase chain reaction (PCR) for pathogens was negative (Table 3).

**Table 3:** CSF analysis findings Tested pathogen in CSF meningitis encephalitis panel: total 14 (*Cryptococcus neoformans/gattii,* *Cytomegalovirus* (CMV), *Enterovirus*, *Escherichia coli *K1, *Haemophilus influenzae*, herpes simplex virus 1(HSV-1), herpes simplex virus 2(HSV-2), human herpesvirus 6 (HHV-6), human parechovirus, *Listeria monocytogenes*, *Neisseria meningitidis*, *Streptococcus agalactiae*, *Streptococcus pneumoniae*, varicella-zoster virus (VZV). CSF: Cerebrospinal fluid, PCR: Polymerase chain reaction

CSF analysis	Result	Reference range
Appearance	Clear, slightly turbid	Clear
WBC count	11 × 10⁶/L (High)	0-7 × 10⁶/L
RBC count	0 × 10⁶/L	0-5 × 10⁶/L
Protein	11 mg/L (Low)	15-40 mg/L
Glucose	78 mg/dL (blood glucose 92 mg/dl, range 60-100mg/dl)	60-80 mg/dL
CSF culture	No growth after five days	
CSF multiplex PCR	Negative for all tested pathogens	

The patient was hospitalized for presumptive mumps encephalitis. Empirical therapy with intravenous ceftriaxone, vancomycin, and acyclovir was initiated. Vancomycin and acyclovir were discontinued once CSF cultures were negative. Ceftriaxone was continued for bacterial coverage. Over 48 hours, her fever and neurological symptoms settled. She reported temporary mild abdominal pain, which subsided spontaneously. By discharge, she was afebrile, hemodynamically stable, and able to consume food and fluids.

## Discussion

Mumps is a vaccine-preventable viral infection that can lead to complications such as encephalitis, meningitis, and orchitis, despite its normal self-limiting nature. Although largely controlled through immunization, sporadic outbreaks continue to occur, raising concerns about prolonged immunity and vaccine coverage [8,9].

Mumps encephalitis occurs in around one in 1000 cases of mumps, and the incidence of severe encephalitis is rare, occurring in one in 6000 cases of mumps [10]. It can follow two pathogenic routes, first by early infection with mumps attacking the CNS directly, and the second is an immune-mediated demyelination, which occurs as a post-infectious complication [11]. In this case, the rapid resolution of the symptoms in 48 hours indicates the direct invasion of the virus into the CNS rather than post-infectious autoimmunity, corresponding with the early encephalitis pathway [11]. Mumps encephalitis is characterized by symptoms such as fever, headache, vomiting, and signs of nervous system involvement, like altered mental status or seizures [12].

This case involves a previously healthy five-year-old girl presenting with classical symptoms of mumps encephalitis. Despite being fully vaccinated as per India’s immunization schedule, she had received the MR vaccine, which does not provide mumps protection. This case contrasts with historical and recent cases of mumps encephalitis in different aspects. Unlike older cases mainly involving unvaccinated individuals with structural damage in the CNS (e.g., hydrocephalus or basal ganglia damage) and prolonged hospitalization, our patient recovered rapidly with supportive treatment without any neuroimaging abnormalities corresponding to a milder presentation in immunocompetent hosts [13,14]. Parotitis was absent in a recent case reported on mumps encephalitis in an adult [15], and the presence of parotitis in this case highlights the variability in clinical manifestations across different cases. Significantly, fatal outcomes were reported with delayed diagnosis or immune-mediated demyelination [16]; our patient’s prompt diagnosis and recovery highlight the improvement with early supportive treatment. These differences highlight that mumps encephalitis remains a diverse entity, affected by immunization status, viral virulence factors, and host immunity. Additionally, even fully vaccinated individuals are not completely protected, as seen in a case of outbreaks among two-dose MMR recipients [17].

Despite high MR vaccine coverage as per India’s UIP, the omission of mumps vaccination leaves children at risk for its potential complications. While MMR is available in private healthcare, its inclusion in the UIP could reduce the risk of encephalitis and potential transmission in the household, as observed in this case, where there was sibling contact [18]. The absence of mumps vaccination also highlights the historical prioritization of MR elimination and cost-effectiveness analyses promoting lower mortality targets [19]. However, complications such as encephalitis and orchitis cause significant morbidity, thereby demanding policy reform [20].

The development from classic mumps symptoms to neurological sequelae reflects the potential severity of mumps encephalitis. Laboratory findings supported a viral etiology, with elevated amylase suggestive of parotitis and mild leukopenia, indicating systemic viral involvement. Despite neurological symptoms being challenging, resolution within 48 hours of supportive care corresponds with the self-limiting nature of mumps encephalitis in immunocompetent hosts.

Early diagnosis is important for managing mumps encephalitis, as highlighted by our case, where the initial symptoms progressed to a serious complication. Distinguishing mumps encephalitis from bacterial or other viral encephalitis (herpes simplex virus) is important. In our case, CSF findings were suggestive of viral encephalitis, supporting a mumps etiology despite a negative CSF pathogen screen result, as our meningitis panel does not screen for mumps virus, positive serum mumps IgM confirmed acute mumps infection. However, while CSF mumps is often negative in mumps encephalitis due to rapid viral clearance from the CNS, serum IgM remains consistent for diagnosis, especially in vaccinated populations where symptoms may be delayed or mild [6]. Neuroimaging (normal CT) ruled out structural abnormalities, underscoring the use of clinical and serological diagnosis.

Supportive care remains the foundation of management, as no specific antiviral therapy exists for mumps. Initial empiric antibiotic and antiviral therapy that was started (ceftriaxone, vancomycin, and acyclovir) was suitably discontinued after excluding the bacterial/herpes simplex virus (HSV) etiologies, emphasizing stewardship in vaccine-preventable diseases. The rapid resolution of symptoms corresponds with literature reflecting favorable outcomes in children without comorbidities.

## Conclusions

This case of mumps encephalitis in an MR-vaccinated child highlights a critical gap in India’s national immunization system. While the MR vaccine has reduced the incidence of measles and rubella, its exclusion of mumps puts children at risk of preventable complications from mumps. The patient’s rapid recovery with supportive care suggests the self-limiting nature of mumps encephalitis in immunocompetent hosts but does not invalidate the need for policy reform.

Introducing the mumps antigen into the UIP would align with the WHO guidelines, prevent complications like encephalitis, and alleviate household transmission risks. This case serves as a call to action. Preventable diseases require preventive solutions. Implementing MMR in India’s UIP could safeguard children from neurological complications and achieve thorough vaccine-preventable disease control.
